# Prefrontal Dopaminergic Regulation of Cue-Guided Risky Decision-Making Performance in Rats

**DOI:** 10.3389/fnbeh.2022.934834

**Published:** 2022-07-11

**Authors:** Minzhe Yang, Qiangpei Fu, Chaolin Ma, Baoming Li

**Affiliations:** ^1^School of Life Science and Institute of Life Science, Nanchang University, Nanchang, China; ^2^School of Basic Medical Sciences and Institute of Brain Science, Hangzhou Normal University, Hangzhou, China

**Keywords:** risky decision-making, dopamine receptors, medial prefrontal cortex, probability, rats

## Abstract

Risky decision-making is the decision made by individuals when they know the probability of each outcome. In order to survive in unpredictable environments, it is necessary for individuals to assess the probability of events occurring to an make appropriate decisions. There are few studies on the neural basis of risky decision-making behavior guided by external cues, which is related to the relative paucity of animal behavioral paradigms. Previous studies have shown that the prefrontal cortex (PFC) plays a key role in risk-based decision-making. The PFC receives projections from the dopamine (DA) system from the ventral tegmental area of the midbrain. The mesocorticolimbic DA system regulates the judgments of reward and value in decision-making. However, the specific receptor mechanism for prefrontal DA regulation of cue-guided risky decision-making behavior remains unclear. Here we established a cue-guided risky decision-making behavioral paradigm (RDM task) to detect the behavior of rats making decisions between a small certain reward and a large uncertain reward in a self-paced manner. The D1 receptor antagonist SCH-23390 (5 mM) or agonist SKF-82958 (5 mM), and the D2 receptor antagonist thioridazine hydrochloride (5 mM) or agonist MLS-1547 (5 mM) was injected into the mPFC, respectively, to investigate how the behavior in the RDM task was changed. The results showed that: (1) rats were able to master the operation of the cue-guided RDM task in a self-paced way; (2) a majority of rats were inclined to choose risk rather than a safe option when the reward expectations were equal; and (3) risk selection was reduced upon inhibition of D1 receptors or stimulation of D2 receptors, but increased upon stimulation of D1 receptors or inhibition of D2 receptors, suggesting that the RDM performance is regulated by D1 and D2 receptors in the mPFC. The present results suggest that DA receptors in the mPFC of rats are involved in regulating cue-guided RDM behavior, with differential involvement of D1 and D2 receptors in the regulation.

## Introduction

Risky decision-making is the decision made by individuals when they know the probability of each outcome. Risk is embodied in the certainty of reward, and the smaller the probability is, the greater the risk will be (Levy et al., 2010). In the process of risky decision-making, individuals need to evaluate the probability and magnitude of each outcome to make the optimal choice (Krain et al., 2006). Individuals respond differently to risk, which leads to different appetites for risk (Dohmen et al., 2005; Rand et al., 2010).

The ability to assess the probability (risk) of the reward relies on a complex neural network that includes the medial prefrontal cortex (mPFC), the orbitofrontal cortex, the nucleus accumbens, and the basolateral amygdala (Mai et al., 2015). Damage to multiple subareas of the prefrontal cortex (PFC), which play key roles in various aspects of decision-making, can lead to decision-making disorders (St Onge and Floresco, 2010; Chau et al., 2018). Patients with damage to the ventromedial and orbital PFC areas are more likely than normal to make risky, adverse choices. The dorsolateral PFC has been implicated in facilitating risk/reward decisions (St Onge and Floresco, 2010).

Dopamine in the brain has been shown to be important in goal-directed behavior, including decision-making (Peters et al., 2020). Dopamine affects behavior by acting on many areas of the brain, such as the PFC (Paul et al., 2007). Due to differences in DA innervation, DA transporters, metabolic enzymes, autoreceptors, receptors, and receptor-coupled intracellular signaling pathways, the role of DA may vary greatly among individuals (Neve et al., 2004; Bentivoglio and Morelli, 2005; Bromberg-Martin et al., 2010). Many studies suggest that the mesocorticolimbic dopamine (DA) system supports risky decision-making (Wise, 2004; Fiorillo, 2011; Orsini et al., 2015; Freels et al., 2020). Genetic variations in dopamine pathways predict risk responses in individuals (Kohno et al., 2016). For example, in a group of adolescent rats with greater risk-taking behavior, lower levels of D2 receptor mRNA expression in the striatum were associated with more self-administration of cocaine in adulthood (Mai et al., 2015). Genetic variations in dopamine transporters predict individual differences in risk selection in capuchins (De Petrillo and Rosati, 2021).

Dopamine receptors are classified into D1 and D2 classes based on their biochemical and pharmacological properties. The two receptors have different signal transduction pathways (Vallone et al., 2000; Berke, 2018). D1 receptors positively regulate the level of cyclic adenosine monophosphate (cAMP), while D2 receptors generally inhibit the activity of adenylate cyclase and reduce intracellular cAMP levels. Both receptors are expressed in the PFC and modulate PFC activity, which is involved in the regulation of cognitive function (Milky et al., 2015; Liu and Kaeser, 2019). However, it remains unclear how differently dopamine receptors in the PFC are involved in regulating cue-guided risky decision-making.

Several tasks have been developed to study the neural basis of risky decision-making in rodents (St Onge et al., 2012; van den Bos et al., 2013). In these tasks, rats associate different actions with contingencies of choice-outcome to generate internal representations of the reward value of each option, which guides their choice behavior. The internal representation of the risk weight may fluctuate from trial to trial as it is affected by the outcome of each choice (van Holstein and Floresco, 2020). As simulated in human behavior paradigms, risky decision-making scenarios in life are often guided by external cues that inform the likelihood of receiving certain rewards (Garofalo et al., 2020). Studies have shown that the manner in which brain circuitry contributes to guiding choice under cue-generated conditions may differ from those where decisions are guided by internally-generated information (Floresco et al., 2018; van Holstein et al., 2020; Schumacher et al., 2021). However, due to the relative paucity of animal behavioral paradigms, there are few studies on the neural basis of risky decision-making behavior guided by external cues (Floresco et al., 2018).

In the present study, we developed a visual cue-guided risky decision-making (RDM) task, where rats chose a safe (small/certain) or a risky (large/uncertain) option in a self-paced manner. Rats made a choice by entering one of the selection arms: the safe arm represented by one light indicated that rats had a 100% chance of getting 50 μl water, while the risk arm represented by two lights indicated that rats had a 33% chance of getting 150 μl water and a 67% chance of getting nothing. The safe and risk visual cues were placed randomly among trials, so rats could not anticipate the choice behavior before the task was initiated. To understand the specific receptor mechanisms for prefrontal DA regulation of cue-guided risky decision-making, we examined changes in risk strategies by modulating the activity of different dopamine receptors in the mPFC.

## Materials and Methods

### Subjects

Thirty-two rats (Sprague–Dawley rats, male, 220–260 g) used in the experiments were kept in the feeding room at Nanchang University Institute of Life Science under standard laboratory conditions. Previous studies showed that a female estrus cycle may influence behavioral performance. In order to obtain stable data, we used male rats for the present study. All the rats were randomly grouped into separate cages after weaning, and each one of them was handled by an experimenter for 5 min/day for 3 days before the pre-training began. To motivate behavior, rats were restricted to water in behavioral sessions and were allowed to drink freely for five minutes after training or testing. Rats had an adequate supply of food every day and had *ad libitum* access to water at least one day per week without participating in training or testing. Rats were trained for 80 trials each daily session. Rats underwent cannulae implantation at 8 weeks after training, and recovered for 1 week. Drug administration was started after their free-choice behavior in the RDM task was stabilized. These rats were divided into vehicle group (8 rats), D1 antagonist group (6 rats), D1 agonist group (6 rats), D2 antagonist group (6 rats), and D2 agonist group (6 rats).

### Behavioral Apparatus

**FIGURE 1:**
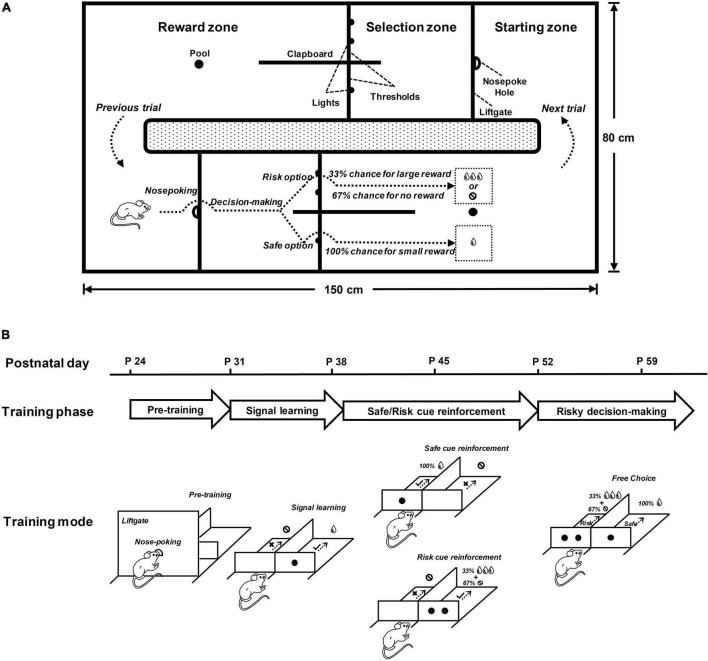
The behavioral paradigm for rats to perform the cue-guided risky decision-making task (RDM). **(A)** This simplified top view shows the main structure of the RDM maze and the performance of a rat in the RDM task. The maze consisted of two identical training chambers placed end to end and was therefore a closed training loop for rats to perform in a naturalistic manner. The upper part of the diagram shows the key structure of the training chamber, outlined with solid lines and texts. The height of the chamber structures was 30 cm, except for the thresholds. The lower part of the diagram shows the behavioral operation of a rat in the training chamber, simulated by the dotted arrows and illustrated by the texts. After completing the operation in one chamber, rats were required to run counter clockwise to the starting zone of the other chamber to start the next trial. **(B)** Learning stages (pre-training, signal learning and safe/risk cue reinforcement) and testing stage (risky decision-making) for the RDM task. The time line shows the approximate points (postnatal day) at which the rats entered the corresponding stages. The simplified diagrams below show the training modes for each stage. In the pre-training stage, the rats were trained to initiate the task by nose poking and to move counter clockwise between the two chambers continuously. In the signal-learning stage, the rats were trained to pay attention to the signal on the threshold in the selection arm. In the safe/risk cue-reinforcement stage, the rats were trained to reinforce cues with rewards. In the risky decision-making stage, the rats were trained to freely make choices between the safe and risky options.

Rats were trained in a maze (150 cm × 80 cm × 30 cm) consisting of two identical training chambers (Figure 1A). The two chambers were connected end-to-end to form a training loop, so rats could use the two chambers alternately in a naturalistic manner to achieve a continuous performance of the task. Each training chamber was composed of three zones in order, namely, the starting zone, the selection zone, and the reward zone. A liftgate (height: 30 cm) with a nose-poke hole in the center was set between the starting zone and the selection zone. Two side-by-side selection arms were set in the selection zone, each with a passable threshold (height: 5 cm) accompanied by lights to indicate cues. A drinking pool was set up in the reward zone, which could store water pumped by the solenoid valve. The behavioral performance in each trial was fed back by audio signals, which were played by speakers at the bottom of the maze. The maze was set in a sound-attenuating room and ran automatically under computer control.

### Behavioral Paradigm

Rats run through the three zones of each training chamber in sequence to perform each trial of the cue-guided RDM task (Figure 1A). Rats were placed in the starting zone of one training chamber and initiated the task performance by probing their noses into the nose-poke hole on the liftgate. The liftgate was lowered (down to 5 cm) for rats to cross into the selection zone within 5 s. The two selection arms in the selection zone were presented with two types of visual cues (on the thresholds), one of which (one light) indicated safe and the other (two lights) indicated risk. Rats made a choice within 5 s according to the cues and crossed the selected arm to enter the reward zone immediately. The speaker played an audio signal based on the performance of the rats, and the solenoid valve pumped the water according to the choice. When rats got a reward at the drinking pool, the liftgate was lifted to its initial position (up to 30 cm) and the chamber temporarily suspended. Rats run counterclockwise to the starting zone of the other training chamber to initiate the next trial. The two training chambers allowed rats to operate alternately to achieve a continuous performance of the RDM task.

In the cue-guided RDM task, the safe cue represented by one light indicated that rats had a 100% chance of getting 50 μl water, and the risk cue represented by two lights indicated a 33% chance of getting 150 μl water and a 67% chance of getting nothing. In each trial, the locations (left or right) for the two cues were randomly assigned. When the performance of rats met the task requirements, the speaker played a high-frequency tone (1000 Hz, 70 dB for 500 ms) to remind rats that their performance was correct. When the performance of rats did not meet the task requirements, the speaker played a low-frequency tone (300 Hz, 70 dB for 500 ms) to remind rats that their performance was wrong. Rats were rewarded with pseudo-random probabilities when choosing a risk option, that is, one out of every three risky choices was rewarded. Figure 1A shows the simulated diagram for the performance of rats on the task, and a video for the real performance of rats on the task was provided in the Supplementary Material.

### Behavioral Training

The cue-guided RDM task included three training stages (pre-training, signal learning, and cue reinforcement) and one testing stage (risky decision-making) (Figure 1B).

#### Pre-training Stage

In the pre-training stage, rats were trained to be aware of initiating the task by making a nose-poking into the hole on the liftgate. To achieve this, a rat was placed in the starting zone of a training chamber. Upon a nose-poking, the liftgate was lowered down to a 5-cm height. The rat ran across the liftgate and moved onto the selection zone and then the reward zone. The rat obtained a water reward at the drinking pool of the reward zone. The liftgate was elevated to a 30-cm height. The rat ran counterclockwise to the other training chamber to initiate the next trial.

#### Signal-Learning Stage

In the signal-learning stage, rats were trained to understand the reward significance of the visual signal (LED light) exhibited on the threshold of the selection arms. After initiating a trial, a rat learned to choose an arm cued by an LED light. The rat could obtain a water reward at the drinking pool of the reward zone if a correct choice was made, or get nothing if an incorrect choice was made. The rat was presented with audio feedback for its choice: a high-frequency tone for a correct choice and a low-frequency tone for a wrong choice. The rat ran counterclockwise to the other chamber to initiate the next trial.

#### Cue-Reinforcement Stage

In the cue-reinforcement stage, rats were trained to understand the different reward significance of the two visual signals: one LED light (safe cue) for a small but definite reward (50 μl water, 100% probability) and two LED lights (risky cue) for a large but non-definite reward (150 μl water, 33% probability). After initiating a trial by nose-poking, a rat came to the selection arms, one of which was indicated by the safe or risky cue. In trials with the safe cue, the rat got a small reward at 100% probability if it chose the cued arm (correct trial), or got nothing if it chose the un-cued arm (error trial). In trials with the risky cue, the rat obtained a large reward at 33% probability if it chose the cued arm (correct trial), or got nothing if it chose the un-cued arm (correct trial). The rat was presented with a high-frequency tone for the correct choice and a low-frequency tone for the wrong choice.

In order for the rat to master the task rule, the training included three training phases in this stage: safe reinforcement, risk reinforcement, and safe/risk reinforcement. In the safe-reinforcement phase, a daily session was composed of safe trials only. In the risk-reinforcement phase, a daily session included risk trials only. In the safe/risk reinforcement phase, a daily session consisted of both safe and risk trials that appeared randomly. Each training phase required the rat to reach an 80% correct performance for three consecutive sessions.

#### Risky Decision-Making Stage

In the risky decision-making stage, rats were trained to make choice decisions between the safe and risky cues. Each daily session in this stage included three blocks of trials, namely, safe-test block (20 trials), risk-test block (20 trials), and free-choice block (40 trials). The performance rules for the safe- and risk-test blocks were the same as described in the cue-reinforcement stage. Only after the selection for the cued arms reached 80% correct, the free-choice block was introduced.

In the free-choice block, after initiating a trial by nose-poking, a rat came to and faced the selection arms, one of which was indicated by the safe cue and the other by the risk cue. The rat got the small reward with a 100% probability if it chose the safe-cued arm or the large reward with a 33% probability if it chose the risk-cued arm.

### Surgery

After the behavioral training, rats underwent surgery under isoflurane anesthesia and were implanted with cannulae (23-gauge, 8.0 mm) for drug infusion in the mPFC (AP: 3.40 mm, ML: ± 0.7 mm, DV: 3.60 mm). The cannulae were fixed with dental cement and secured with skull screws. Tube plugs were inserted into the cannulae to prevent brain tissue from infection.

### Drug Administration

Four dopaminergic drugs were used in the present study, namely, the D1 receptor agonist SKF-82958 (5 mM) and antagonist SCH-23390 (5 mM); the D2 receptor agonist MLS-1547 (5 mM) and antagonist thioridazine hydrochloride (5 mM), which were purchased from Sigma–Aldrich, United States. Each drug was dissolved in a small volume of dimethyl sulfoxide (DMSO, Sigma-Aldrich) and diluted with sterile saline (final DMSO concentration was 5%). The vehicle was saline containing 0.5% DMSO. A drug solution or vehicle was bilaterally infused into the mPFC at a dose of 0.5 μl 15 min before behavioral testing was conducted. For infusion of drug solution or vehicle, the tube plugs were removed from the cannulae, and infusion needles were inserted into the cannulae. The infusion needles were connected to 5-μl micro-syringes via polyurethane tubes. The infusion rate was 0.2 μl/min. For the rats in the drug groups, bilateral double intra-mPFC infusions were conducted at the same site to confirm the effects of the drugs. The second dose was administered after the effect of the first dose had worn off and when the behavioral performance of the rats had returned to baseline level. A bilateral single intra-mPFC infusion was conducted for the rats in the vehicle group.

### Histology

To verify the location of the infusions, rats were anesthetized with isoflurane and perfused transcardially with saline and 4% paraformaldehyde (PFA). Brains were removed and submerged into a sucrose solution to dehydrate. The brains were then sectioned with a freezing cryostat (Leica CM1950, Germany) at a 30-μm thickness. Brain sections were mounted and stained with Nissl staining. The infusion sites were examined under a light microscope. The anterior-posterior position range of the cannulae placements was 3.00–3.90 mm anterior to the bregma.

### Data Acquisition and Analysis

Data were analyzed with IBM SPSS Statistics 25 and expressed as mean ± SEM. For the signal-learning and cue-reinforcement stages, the frequency of choosing the signal or cued-arm (selection arm with signal or cue) was calculated, and variables across different sessions were tested using one-way ANOVA with the Tukey test for multiple comparisons to determine whether the performance had reached a steady state. For the risky decision-making stage, the frequency of choosing the cued arm was calculated in the safe- and risk-test blocks, and the frequency of choosing the risk arm (selection arm with risk cue) was calculated in the free-choice block. The baseline performance of risk selection for each rat was the average of its stable performance over the three consecutive daily sessions prior to surgery. The so-called “stable performance” was defined as having no significant difference among the three prior-surgery sessions. A paired sample *t*-test was used to compare the vehicle effects on the performance of the rats. Repeated measures one-way ANOVA (1R-ANOVA) with the Bonferroni test for *post hoc* analyses were used to compare the performance of the rats before and after dopaminergic drug administration.

## Results

### Rats Preferred to Choose Risk Under Equal Expectation Condition

**FIGURE 2:**
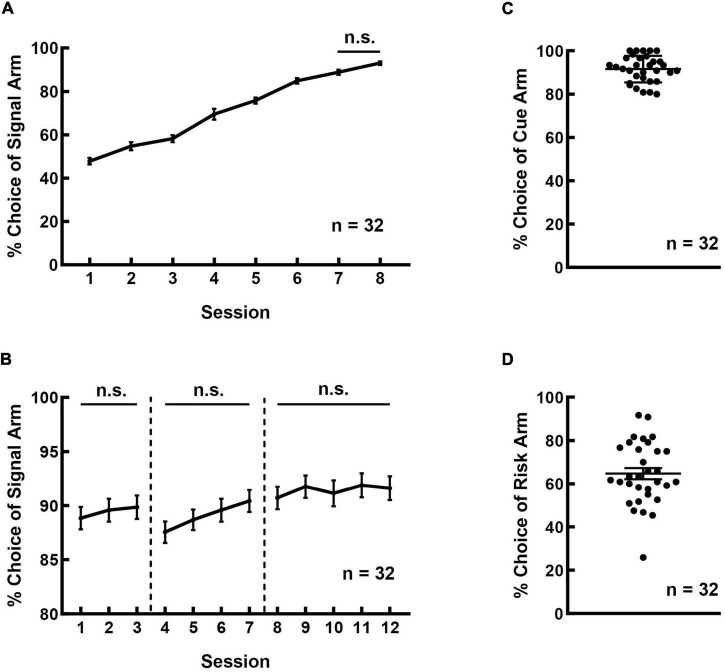
Rats could learn the cue-guided RDM task and showed a preference for the risk option. **(A)** The learning curve of the rats during the signal-learning stage. **(B)** The learning curve of the rats during the cue-reinforcement stage. The safe-cue reinforcement, risk-cue reinforcement, and random reinforcement phases are separated by the dotted vertical lines. **(C)** The frequency of choosing a cued arm in the cue-test blocks was higher than 80%. **(D)** A majority of rats exhibited preference for the risk arm in the free-choice block.

The rats were able to master the cue-guided RDM task through two-month of training. Figure 2A shows the learning curve of the rats in the signal-learning stage. The rats could recognize the significance of LED signals via one-week training. The frequency of choosing the signal arm reached over 80% in the 6th session and kept stable during the last two consecutive daily sessions (*F*_(7_, _255)_ = 112.580, *p* < 0.001; Session 6 vs. 7: *p* = 0.652; Session 7 vs. 8: *p* = 0.559; other pairs: *p* < 0.05).

Figure 2B shows the learning curve of the rats in the cue-reinforcement stage. The three phases of this learning stage (safe-cue reinforcement, risk-cue reinforcement, and random reinforcement) are separated by the dotted vertical lines. As shown, the frequency of choosing cued arm remained high and had no significant difference among the phases (Figure 2B; safe: *F*_(2_, _95)_ = 0.246, *p* = 0.782; risk: *F*_(3_, _127)_ = 1.518, *p* = 0.213; random: *F*_(4_, _159)_ = 0.195, *p* = 0.941).

Figure 2C shows the performance of the rats in the risky decision-making stage. The rats performed well in the safe- and risk-test blocks and the frequency of selecting cued arms was 91.562 ± 1.086%. While in the free-choice block, the rats displayed marked individual variability in their preference for the large and risky reward. The rats exhibited no significant differences in the frequency of choosing risk options among the last three consecutive daily sessions (*F*_(2_,_95)_ = 0.012, p = 0.988). Significant correlations were detected among these three sessions (Session 1&2: *r* = 0.986, *p* < 0.001; Session 2&3: *r* = 0.985, *p* < 0.001; Session 1&3: *r* = 0.980, *p* < 0.001). A majority of rats preferred the risk arm, with an average choice frequency of 64.700 ± 2.557% (Figure 2D).

### Prefrontal Cortical D1 Receptors Are Involved in Risky Decision-Making

**FIGURE 3:**
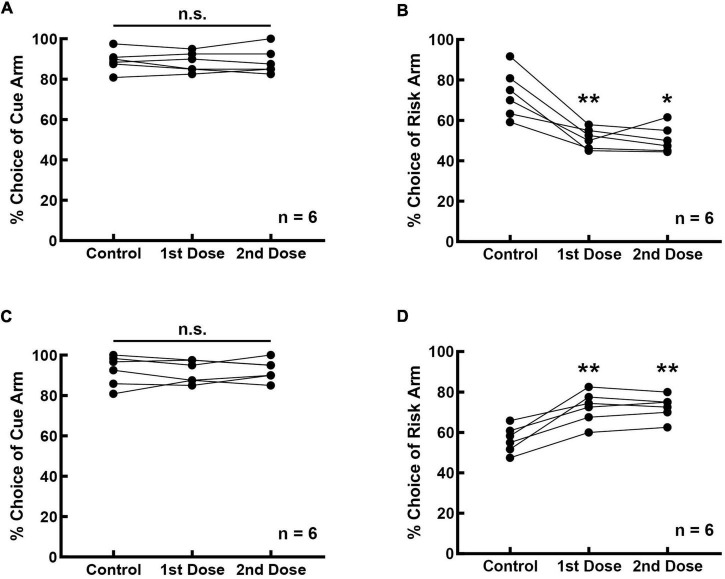
Prefrontal D1 receptors are involved in regulating cue-guided RDM behavior. **(A)** Bilateral infusion of the D1 antagonist SCH-23390 into the mPFC did not affect rats’ performance in the cue-test blocks. **(B)** Risk selection in the free-choice block was reduced upon inhibition of D1 receptors in the mPFC. **(C)** Bilateral infusion of the D1 agonist SKF-82958 into the mPFC did not affect rats’ performance in the cue-test blocks. **(D)** Risk selection in the free-choice block increased upon stimulation of D1 receptors in the mPFC. *p < 0.05; **p < 0.01.

The effect of the D1 receptor activity on risk-choosing strategies was investigated in 12 rats, six of which were infused with D1 receptor agonists and the remaining six rats with D1 receptor antagonists. Bilateral intra-mPFC infusion of the D1 antagonist SCH-23390 did not affect the accuracy of performance in the cue-test blocks (Figure 3A, *F*_(2_,_10)_ = 0.195, *p* = 0.826). It was 89.167 ± 2.205% before SCH-23390 infusion, and 88.333 ± 2.007% (1st dose) and 88.750 ± 2.642% (2nd dose) after infusion. However, inhibition of prefrontal D1 receptors reduced the choice of the rats for the risk arm (Figure 3B, *F*_(2_, _10)_ = 21.080, *p* < 0.001). The frequency of selecting the risk arm was 73.333 ± 4.854% before the infusion, and 51.091 ± 2.051% (1st dose) and 50.559 ± 2.702% (2nd dose) after the infusion. Infusion with SCH-23390 reduced the frequency of risk choice by 22.509% on average (22.242% for the 1st dose, *p* = 0.009; 22.775% for the 2nd dose, *p* = 0.018).

Bilateral intra-mPFC infusion of the D1 agonist SKF-82958 into the mPFC also did not affect the accuracy of performance in the cue-test blocks (Figure 3C, *F*_(2_, _10)_ = 0.158, *p* = 0.856). The frequency of cue selection was 92.361 ± 3.099% before the SKF-82958 infusion, and 91.667 ± 2.297% (1st dose) and 92.500 ± 2.141% (2nd dose) after the infusion. However, stimulation of prefrontal D1 receptors increased the choice of the rats for the risk option (Figure 3D, *F*_(2_, _10)_ = 31.982, *p* < 0.001). The choice frequency of the risk arm was 56.528 ± 2.682% before the infusion, 72.393 ± 3.213% (1st dose) and 72.500 ± 2.415% (2nd dose) after the infusion. Infusion with SKF-82958 increased the choice frequency of the risk arm by 15.919% on average (15.865% for the 1st dose, *p* = 0.009; 15.972% for the 2nd dose, *p* = 0.004).

### Prefrontal Cortical D2 Receptors Are Involved in Risky Decision-Making

**FIGURE 4:**
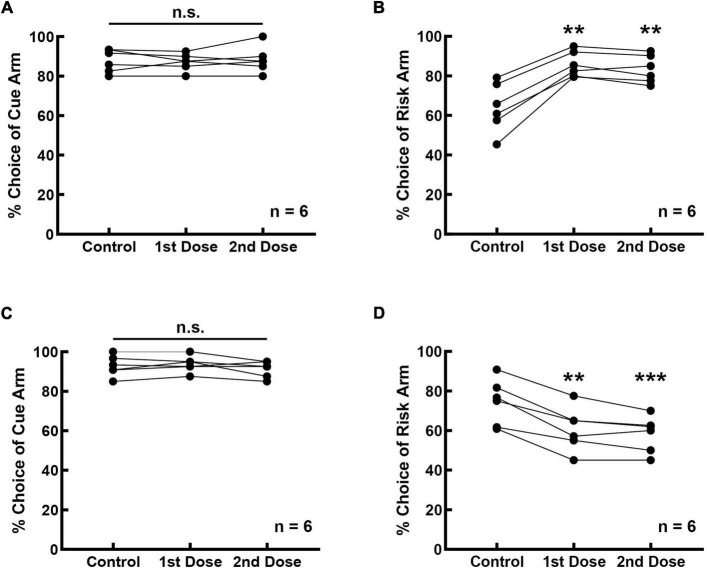
Prefrontal D2 receptors are involved in regulating cue-guided RDM behavior. **(A)** Bilateral infusion of the D2 antagonist thioridazine into the mPFC did not affect rats’ performance in the cue-test blocks. **(B)** Risk selection in the free-choice block was increased upon inhibition of D2 receptors in the mPFC. **(C)** Bilateral infusion of the D2 agonist MLS1547 into the mPFC did not affect rats’ performance in the cue-test blocks. **(D)** Risk selection in the free-choice block was reduced upon stimulation of D2 receptors in the mPFC. **p < 0.01; ***p < 0.001.

The effect of the D2 receptor activity on risk-choosing strategies was investigated in 12 rats, six of which were infused with the D2 receptor agonist and the other six with a D2 receptor antagonist. Bilateral intra-mPFC infusion of the D2 receptor antagonist thioridazine did not affect the cue recognition of the rats (Figure 4A, *F*_(2_, _10)_ = 0.333, *p* = 0.725). The frequency of choosing the cue arm was 87.778 ± 2.373% before thioridazine infusion, and 87.083 ± 1.758% (1st dose) and 88.333 ± 2.713% (2nd dose) after the infusion. However, inhibition of prefrontal D2 receptors increased selection for risk (Figure 4B, *F*_(2_, _10)_ = 40.720, *p* < 0.001). The frequency of selecting risk option increased from 64.092 ± 5.073% (control) to 85.743 ± 2.639% (1st dose) and 83.374 ± 2.882% (2nd dose), with an average increase of 20.467% (21.651% for the 1st dose, *p* = 0.002; 19.282% for the 2nd dose, *p* = 0.007).

Bilateral intra-mPFC infusion of the D2 agonist MLS1547 did not affect the accuracy of performance in the cue-test blocks (Figure 4C, *F*_(2_, _10)_ = 2.170, *p* = 0.165). The frequency was 92.778 ± 2.126% before the infusion, 93.750 ± 1.677% (1st dose) and 91.250 ± 1.677% (2nd dose) after the infusion. However, stimulation of prefrontal D2 receptors reduced the selection of the rats for risk option (Figure 4D, *F*_(2_, _10)_ = 57.514, *p* < 0.001), from 74.444 ± 4.742% (control) to 60.774 ± 4.512% (1st dose) and 58.234 ± 3.725% (2nd dose), with an average reduction of 14.941% (13.671% for the 1st dose, *p* = 0.003; 16.210% for the 2nd dose, *p* < 0.001).

### Intra-MPFC Infusion of the Vehicle Did Not Affect Risky Decision-Making

**FIGURE 5:**
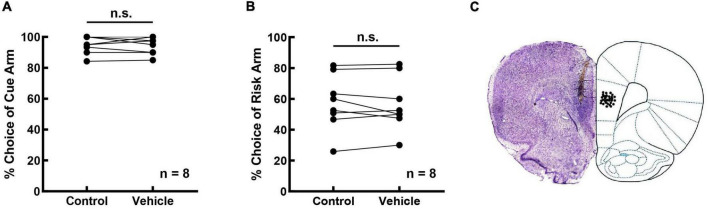
Intra-mPFC infusion of vehicle did not affect risky decision-making. **(A)** Vehicle did not affect the cue recognition ability of rats when tested in the cue-test blocks. **(B)** Vehicle did not affect the risk strategies held by rats when tested in the free-choice block. **(C)** An example for the location of drug infusion in the mPFC. Brain sessions were stained with Nissl’s method.

The possible effect of a vehicle on risk-choosing strategies was investigated in eight rats. As shown in Figure 5, bilateral intra-mPFC infusion of vehicle did not affect the cue recognition of the rats (Figures 5A; R = 0.829, *p* = 0.011; *t* = 0.273, *p* = 0.793). The frequencies of cue selection before and after the infusion were 94.687 ± 1.979% and 94.375 ± 1.934%, respectively. Also, the vehicle had no effect on the risky strategies of the rats (Figures 5B; *R* = 0.964, *p* < 0.001; *t* = 0.552, *p* = 0.598). The frequency of risk selection was 57.500 ± 6.386% before the infusion vs. 56.563 ± 6.159% after the infusion. The infusion sites were examined using Nissl’s staining, and an example of an infusion site (3.72 mm anterior to the bregma) is shown in Figure 5C.

## Discussion

In the present study, we used a cue-guided RDM behavioral paradigm to examine prefrontal dopaminergic regulation of risky decision-making in rats. The rats developed stable decision strategies after mastering the rules of the task. As seen from their selection in this task, the rats had different responses to risks and tended to choose the risk option. Inhibition or stimulation of D1 and D2 receptors in the mPFC produced opposite effects on risky decision-making in rats.

The cue-guided RDM task has many characteristics that previous tasks do not have, and could meet many research needs: (1) rats are guided by visual cues to make decisions in a more naturalistic manner, choosing between paths over levers; (2) the behavioral motivation of rats is generated by water restriction, under which rats experience less stress (Tucci et al., 2006; Goltstein et al., 2018); (3) rats operate the RDM task continuously in two connected training chambers, which avoids the location preference; (4) behavioral operation is reinforced by audio feedback, which helps rats to make the goal-directed decision; and (5) the visual cues guiding the decision are presented after the initiation of the trial, which restricts the decision-making behavior to a specific time period and was conducive to the study of the neural mechanisms underlying risky decision-making in rats.

The behavioral training of the RDM task took more than a month, starting at P24 through P60 approximately. Previous studies have shown that the expression of dopamine receptors and the synergy of D1/D2 receptors in the mPFC of rats are age-dependent (Dwyer and Leslie, 2016). In the present study, dopaminergic manipulation in the mPFC was conducted after the rats had mastered the task performance when the rats were already adults. Also, the training procedures of the RDM task would prevent the locomotor activity and memory of the animals from declining (Shoji et al., 2016).

The present study shows that the risk selection of rats decreased upon inhibition of prefrontal D1 receptors but increased upon stimulation of prefrontal D1 receptors. This result is consistent with previous studies showing that viral overexpression of dopamine D1 receptors in the mPFC was associated with increases in drug seeking, impulsivity, and hedonic behavior in adult rats (Kai et al., 2014; Freund et al., 2016). Beyer et al. (2021) reported that subjects with overexpression of D1 receptors exhibited maladaptive risky decision-making (choosing high-risk and high-reward options), and the risky decision returned to the control level after the termination of D1 receptor overexpression.

On the contrary, the present study found that manipulation of prefrontal D2 receptors produced the opposite effect. Risk selection of the rats increased upon inhibition of prefrontal D2 receptors but increased upon stimulation of prefrontal D2 receptors, consistent with the previous study by St. Onge et al. showing that blockade of D2 receptors increased risky choice and the study by Simon et al. showing that stimulation of D2 receptors robustly attenuated risk-taking (Simon et al., 2011; St Onge et al., 2011). However, the effects caused by the stimulation of D2 receptors are controversial. St Onge et al. (2011) found that stimulation of D2 receptors induced a true deficit in decision making. Such inconsistency might be due to the difference in the behavioral paradigms. St. Onge et al. adopted the probability discounting task, where the probability of reward changed in each daily session. Rats associated different actions with choice-outcome contingencies to generate internal representations for action-reward associations. It might be possible that the behavior of their rats reflected not only risk strategy but also risk perception. In addition, the change in reward probability would lead to a change in reward expectation, which involves both risk strategy and value strategy in choices.

In the present study, we found that different dopamine receptors play different roles in regulating the cue-guided risky decision-making strategies, as in previous studies using a decision by internal representation. Unbalanced activation of D1 vs. D2 receptors in the PFC may lead to abnormal prefrontal function (Victoria and Miller, 2015). Nicole et al. proposed that D1 and D2 receptors facilitate diverse aspects of decision-making by acting on separate networks of prefrontal neurons that interface with distinct targets such as the striatum and amygdala (Jenni et al., 2017). Dopamine receptors have been classified based on their biochemical and pharmacological properties, and there are abundant expressions of dopamine subtypes in the mPFC. Thus, it is necessary to further clarify the roles of each receptor subtype in cue-guided risky decision-making.

The PFC is known to be highly sensitive to its neurochemical state given by the diffuse ascending inputs from dopamine, norepinephrine, serotonin, and acetylcholine neurotransmitter systems. It has been reported that PFC-mediated cognitive functions could be influenced by dopamine in a non-linear fashion (Robbins, 2010). For instance, Nicholas et al. reported that the expression of D2 receptor mRNA in mPFC predicted risk preference in opposing non-linear patterns (Simon et al., 2011). Susheel et al. showed that stimulation of D1/D5 receptors in the PFC produces an ‘inverted-U’ dose-response on working memory function, whereby either too low or too high stimulation impairs PFC-dependent cognitive performance (Vijayraghavan et al., 2007). Hence, it is necessary to further study if the dopamine system also plays a non-linear role in regulating cue-guided risky decision-making, so as to understand how dopamine affects the mPFC to influence cue-guided risky decision-making behavior more comprehensively.

## Data Availability Statement

The original contributions presented in this study are included in the article/Supplementary Material, further inquiries can be directed to the corresponding author/s.

## Ethics Statement

The animal study was reviewed and approved by Medical Laboratory Animal Ethics Committee of Nanchang University.

## Author Contributions

MY: methodology, software, validation, formal analysis, investigation, data curation, writing – original draft, and visualization. QF: validation, formal analysis, investigation, and visualization. BL and CM: conceptualization, methodology, resources, data curation, writing – review and editing, supervision, project administration, and funding acquisition. All authors contributed to the article and approved the submitted version.

## Conflict of Interest

The authors declare that the research was conducted in the absence of any commercial or financial relationships that could be construed as a potential conflict of interest.

## Publisher’s Note

All claims expressed in this article are solely those of the authors and do not necessarily represent those of their affiliated organizations, or those of the publisher, the editors and the reviewers. Any product that may be evaluated in this article, or claim that may be made by its manufacturer, is not guaranteed or endorsed by the publisher.
